# On the Possibility of Fluorescent Capture Immunoassays on a Contact Lens

**DOI:** 10.3390/bios15050326

**Published:** 2025-05-20

**Authors:** Kundan Sivashanmugan, E. Albert Reece, Joseph R. Lakowicz

**Affiliations:** 1 Center for Fluorescence Spectroscopy, Department of Biochemistry and Molecular Biology, University of Maryland School of Medicine, 721 West Lombard St., Baltimore, MD 21201, USA; skundan@som.umaryland.edu; 2 Department of Obstetrics, Gynecology and Reproductive Sciences, University of Maryland School of Medicine, 655 W., Baltimore, MD 21201, USA; areece@som.umaryland.edu

**Keywords:** contact lens, silicon hydrogel lenses, lysozyme, biomarker detecting, tears, fluorescein

## Abstract

Blood samples and testing are routine in healthcare. Presently, there is a growing interest in using tear samples in place of blood. Tear samples can be obtained non-invasively and collection does not require the skills of a trained phlebotomist. Red blood cells and other cells are not present in tears, which avoids centrifugation. Importantly, basal tear samples contain most of the biomarkers present in blood. The difficulty is the small volume of basal tears, which is about 7 μL in each eye. Any contact with the eye results in additional reflex tears with a different chemical composition. The small tear samples are collected with capillary tubes and then sent out for amplified assays, such as enzyme-linked immunosorbent assay (ELISA) or polymerase chain reaction (PCR). The results are not available for several days or a week and, therefore, are less useful in an ophthalmology office. We propose the use of a contact lens that contains bound antibodies for fluorescence immunoassays. The lenses could be removed from the patient for point-of-care measurements at the bedside. To prove that this concept is possible, we performed a three-layer protein capture assay that mimics an immunoassay. For convenience, we used lysozyme (Lys), which spontaneously coats silicon hydrogel (SiHG) contact lenses (CL). Anti-lysozyme IgG was the second layer captured, with anti-lysozyme considered to be the target biomarker. The third layer was rhodamine or Alexa Fluor-labeled Ab against the IgG Fc region, considered to be the detection antibody. The multiple protein layers were stable and did not wash off the SiHG lenses. These results strongly suggest the contact lens can be used for capture immunoassays for a wide variety of biomarkers.

## 1. Introduction

Blood samples are used for diagnostic testing [1,2]. Apart from conventional electrolyte panels, specific assays are used to detect a wide variety of biomarkers linked to heart disease, bladder disease, lung disease, pancreatic cancer, breast cancer, and diabetic retinopathy [3,4,5,6,7,8]. The list of known biomarkers in blood is extensive [9,10,11,12], and additional novel biomarkers are frequently reported [13,14]. Other fluids are used for biomarker diagnostic purposes including urine, saliva, and mucosal secretions [15,16,17,18,19,20,21]. Saliva is known to possess proteases that have the capability to degrade some biomarkers. The use of urine biomarkers for prostate cancer detection is influenced by the pH of the urine, which may impact the observed concentrations of several other biomarkers [22,23]. There have been advances in biomarker detection methods, such as surface plasmon resonance (SPR), 2D chromatography, enzyme-linked immunosorbent assay (ELISA), plasmonic nanoparticles, and liquid chromatography with tandem mass spectrometry (LC-MS/MS) [15,16,17,18,19].

Venous blood samples are considered the most efficient approach for identifying biomarkers and conducting blood tests [24,25]. The procedure of extracting blood samples from a vein can be complicated, mostly linked to the following challenges: (i) access to veins is a complex task that requires the participation of skilled healthcare professionals; (ii) the act of collecting blood samples requires clinical ability and adherence to sterile procedures; (iii) other factors can complicate the findings, such as the patient’s age and weight; and (iv) errors in handling the blood can occur, including clotting, inaccuracies in dilution, and variations in biomarker levels [24,25,26,27,28]. The factors mentioned above are referred to as pre-analytical variability and are believed to be responsible for approximately 90% of diagnostic errors [27]. These problems are even more difficult for lumbar punctures, which require patient sedation. The collection of blood samples using needles is convenient, but there is a need for improved methods of collecting body fluid samples for diagnostics.

Collecting tear fluid is a non-invasive method, and tear samples contain a wide range of biomarkers, potentially including all those found in blood [29,30,31]. According to Mitalee et al., there are more than 100 distinct biomarkers that may be found in tear fluid [29]. A limiting factor in using tears is the difficulty of obtaining an unperturbed sample. The total volume of tears for one eye is about 7 μL [32,33]. Tear samples are commonly acquired by the use of capillary tubes or adsorbent paper strips similar to those used to measure tear output. The samples are sent out to measure electrolyte concentrations or specific biomarker testing [34,35]. Importantly, the eyes are sensitive and any physical contact results in a rapid physical change in the type of tears produced [36,37]. Basal tear fluid, reflex tears, and emotional tears each have a different composition of proteins [38,39]. Because of the small tear volumes available for analysis, the biomarker assays are performed using highly sensitive methods such as ELISA, SPR, fluorescence, electrochemical methods, LC-MS/MS, and nanosensors [40,41,42].

**Figure 1:**
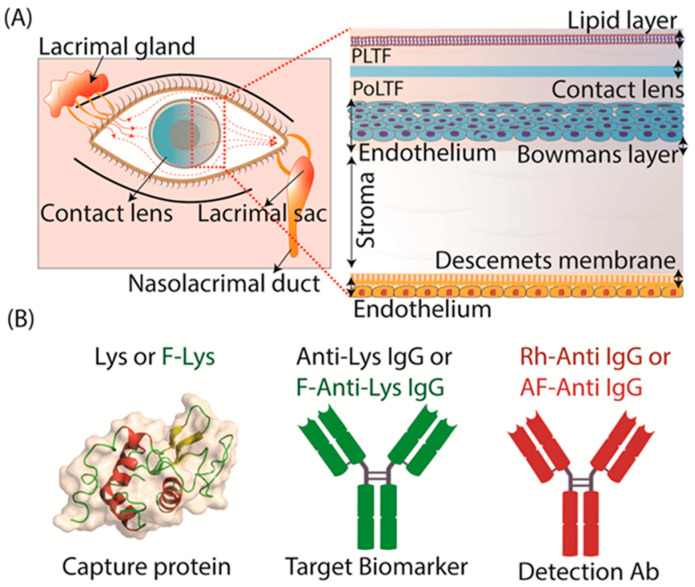
(**A**) The cornea’s structure with a contact lens (blue) localized in the center aqueous portion of the tear film. (**B**) Role of protein used in the present paper.

Currently, there is a growing trend towards the use of tear fluids for different applications. The identification of biomarkers in tear fluids is a potential alternative to blood-based biomarker detection [30,43,44,45,46]. Because tears do not contain red blood cells and most other circulating cells are removed from tears, the samples are already partially purified. Tear films consist of numerous components that create a physical barrier against particles, bacteria, and viruses [43,44,45,46]. Tear film, a thin extracellular layer, protects and cleans the eye. It is typically 3–40 µm thick, secretes at a rate of 1.2 µL/min, and has a volume of 5–10 µL [47,48]. The eye’s tear surface film is made up of three layers: middle aqueous, outer lipid, and inner mucin (Figure 1A). It is composed of water, salts, proteins, lipids, and mucins. The protein makeup of the aqueous layer changes with eye state (open/closed) and tear type (reflex/basal) [47,48]. Tear films are provided by the constant production of fresh tear fluid by continuous secretions from the lacrimal, Meibomian, and reflex glands. Except for a possible short time delay, the tears are in rapid equilibrium with blood [46,49,50]. Tears could be used for biomarker detection if appropriate technology is made available for the samples. The processing of the tear samples for such measurements is time consuming and requires sophisticated apparatus [31,51]. Consequently, such measurements are not currently applicable for point-of-care applications.

Contact lenses (CLs) have many beneficial characteristics for biosensing applications [52,53,54]. In addition, CLs establish direct touch with ocular tears and the corneal epithelium. CLs are considered a minimally invasive technologies; are small, light, inexpensive, and portable; and may include a variety of sensors [54,55,56,57,58,59]. The close connection of the many components of the eye and CLs made it possible to develop lenses that can serve as continuous monitoring platforms. Recent studies demonstrated a correlation between glucose levels in tears and blood glucose level with a delay of 10–20 min. In recent times, multicolor nanozyme-infused cosmetic CLs have been produced to prevent ocular surface diseases. This is accomplished by incorporating Prussian blue nanoparticles into the lens matrix, allowing the catalytic scavenging of reactive oxygen species [60]. Also, a hemispherical system with Ti_3_C_2_T_x_ MXene has been used for intraocular pressure monitoring. This method combines a micro-supercapacitor (MSC) with strain sensors to produce self-supporting devices [61,62]. The two types of sensor-integrated CLs have high sensitivity, despite the fact that they need several fabrication procedures.

Multiple groups have worked on embedding sensors into contact lenses. Google attempted to make a glucose-sensitive contact lens using embedded electronics [53]. This lens used the standard approach with glucose oxidase, electrochemical detection, and radio frequency induction for power (Figure S1). The GlucoLens was announced in 2014 with participation by Novartis, Alphabet, and other companies [52,53,54,55,56,57,58,59]. The GlucoLens project was dropped in November 2018. Another example is Mojo Vision, Inc., which started work on a more advanced smart contact lens in July 2017 and, by July 2022, had support from 32 companies [52,53,54,55,56,57,58,59]. The Mojo lens was promised to display augmented reality to the wearers. This project was abandoned in January 2023, but Mojo announced new funding to make smart contact lenses [52,53,54,55,56,57,58,59]. Other companies have developed contact lenses to detect glaucoma or for image display (Figure S1). However, all of these methods require embedding components into the lenses. Few, if any, of these projects resulted in a final product. These commercial and private efforts demonstrate the difficulty of placing the needed electronics or photonic components within a contact lens. If one of these electronic lenses was successful and designed for long-term wear, the components would need to be embedded in a wide variety of contact lens polymers for different patients.

Our approach to making a sensing or biomarker contact lens (BM-CL) is very different from previous efforts. We do not place any electronics or specialized optical components within the lens (Figure S2). The only active components in our BM-CL are fluorescent proteins bound to the contact lens. This approach can be used with most contact lenses in current use, but our experiments found the silicone hydrogel (SiHG) lenses to be most suitable and SiHG lenses are widely used and acceptable to most patients [63,64,65,66]. The extensive publication library on binding proteins to surfaces can be used in the fabrication of these lenses. Biomarker identification is facilitated by the increasing number of recombinant mono-cloned antibodies that have been developed by industry and private laboratories [67,68,69]. Our approach to surface-labeled contact lenses allows applications beyond biomarkers and extends to most of the electrolytes in tears and glucose [63,64,65,66]. Our unique approach also allows separate measurements on each side of a contact lens, the pre-lens tear film (PLTF) and the post-lens tear film (PoLTF) [66], which may allow selective observation of ocular surface diseases on sensitive epithelia corneal cells. In this work, we developed a fluorescent immunoassay for the detection of lysozyme (Lys) on the surfaces of CLs. Notably, Lys is an antibacterial enzyme found in different bodily fluids, including tears and saliva. Lys in tears is essential for protecting the eyes from bacterial infections. The physiological concentration of Lys in tears is around 1.33 mg/mL, with some studies suggesting a range of 1.4 to 2.1 mg/mL [70,71]. Consequently, the detection of Lys is straightforward due to its higher content in tears. CLs underwent the first Lys coating procedure. To measure fluorescence, a fluorescence immunoassay was established for the lenses, which examined Lys uptake and the interaction of primary and secondary antibodies on the lens surface. It is expected that BM-CLs will ultimately serve as a specific coating or antibody conjugation designed to target and identify a wide range of biomarkers.

## 2. Materials and Methods

We used widely available proteins and antibodies to develop a contact-lens-based pseudo-immunofluorescence assay. In this study, we used lysozyme (Lys) (Cat. No. 89833, MW 14.38 kDa) and fluorescein-labeled lysozyme (F-Lys) (Cat. No. LS1-FC-1) from ThermoFisher Scientific (Chicago, IL, USA) and Nanocs (New York, NY, USA), respectively. Lys binds spontaneously to SiHG lenses, and we considered Lys or F-Lys to be the capture protein. We obtained the primary antibodies from Abcam (Waltham, MA, USA): unlabeled anti-lysozyme antibody (Anti-Lys) (Clone No. EPR2994(2), host-Mouse) and fluorescein-conjugated anti-lysozyme antibody (F-anti-Lys) (Clone No. LZ598-10G9, host-Mouse) (Figure 1B). The F-Lys Ab was the target antigen to be captured. As detection antibodies, rhodamine red-labeled goat anti-rabbit IgG (Rh-anti-IgG) (Cat. No. R-6394) and Alexa Fluor 647-labeled goat anti-human IgG (AF-anti-IgG) (Cat. No. A-21445) were obtained from ThermoFisher Scientific (Chicago, IL, USA). Phosphate-buffered saline (PBS), pH 7.2, was used for sample dilution and fluorescence measurements throughout this work.

### 2.1. Selection of Silicone Hydrogel (SiHG) Contact Lens

We selected commercially available and widely used silicone hydrogel (SiHG) Lotrafilcon A (Lot A) (Air Optix lenses from Alcon, Elkridge, MD, USA) contact lenses and Comfilcon A (ConA) Bioaffinity lenses from Cooper Vision. The SiHG lenses exhibited distinct chemical and physical characteristics, as detailed in the Supplementary Materials (Table S1). SiHG lenses contain continuous regions of silicone from the front to the back of the lens, and both are expected to have interface regions for hydrophobic binding (Figure S3). Notably, about 65% of new prescriptions are for these SiHG lenses [72,73]. For comparison, Table S1 also lists properties for a standard hydrogel (HG) lens. HG lenses exhibit lower oxygen transport (Dk) and are worn for shorter periods of time. In previous reports, we showed that SiHG lenses can bind either hydrophobic species or highly charged poly-lysine [63,64,65,66]. In the present report, we use lysozyme, which has a high positive charge and binds strongly to SiHG lenses. The experiments in this report were performed using the Lot A lenses, but similar results were obtained with the ConA lenses.

### 2.2. Fluorescence Measurements

Fluorescence measurements were performed using a photon-counting spectrofluorometer, the FluoTime 300 from PicoQuant, GMBH. A pulsed laser diode was used at an excitation of 470 nm at 40 MHz using an amplified fiber laser for excitation at 532 nm. Some emission spectra and intensities were measured using a Varian Cary Eclipse 4 spectrofluorometer. Intensity and lifetime images of the lenses, and scans across the central axes of the lenses, were performed using an Alba 5 time-resolved laser scanning confocal microscope from Alba ISS Inc. (Champaign, IL, USA). Excitation was conducted at 473 nm using a 20× objective. A 25 nm pinhole was used for confocal measurements. The observed size was approximately 0.5 mm in diameter with 256 × 256 pixels. The scan across the lenses was measured at intervals of 0.5 mm over the 13 mm lens diameter, with the focus of the objective lens adjusted to the height (z-axis) location in the lens.

## 3. Results

### 3.1. SiHG Lens Selection and Lysozyme Binding to Lenses

**Figure 2:**
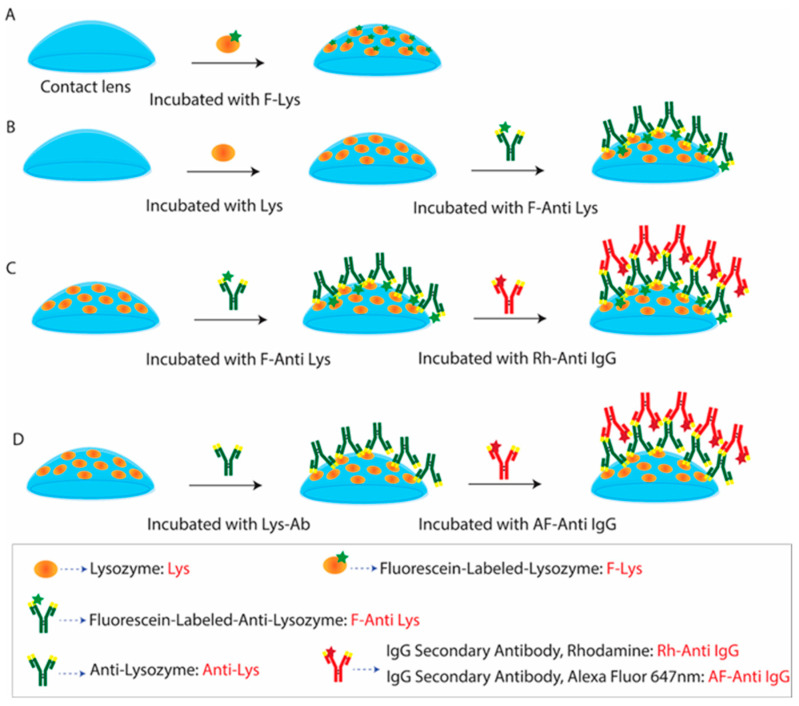
The experimental design used for the detection of Lys or F-Lys binding Lot A lenses by fluorescence measurement. (**A**) F-Lys binding to lens, (**B**) F-Anti Lys binding to Lys-lens, (**C**,**D**) detection of F-Anti Lys or anti Lys on lens using secondary Ab (Rh-Anti IgG or AF-Anti IgG).

**Figure 3:**
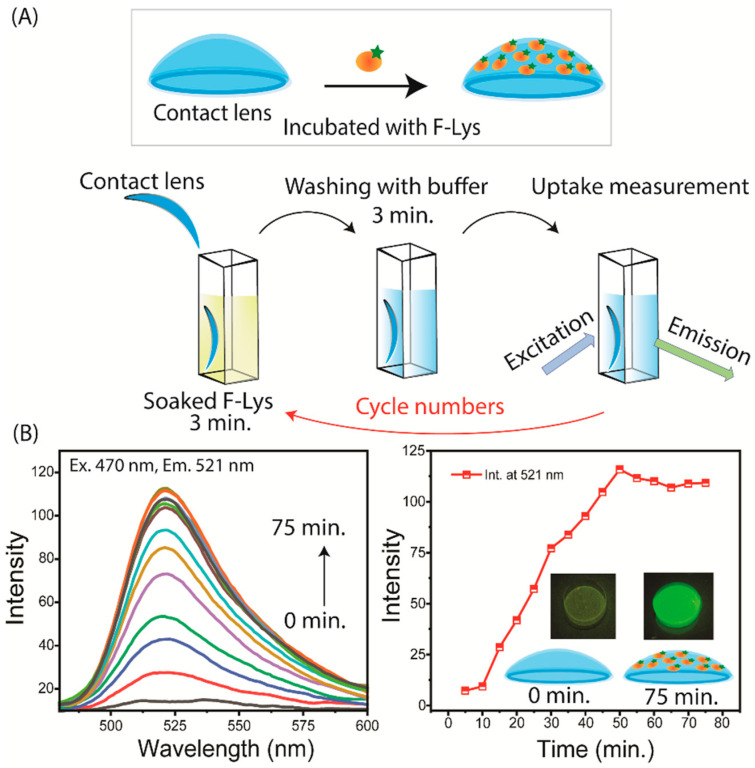
(**A**) The procedure used to prepare F-Lys coated on a Lot A lens: every three minutes, a lens sample is incubated with F-Lys, subsequently washed with buffer solution, and the emission spectra are measured using a spectrofluorometer. The holder containing the fixed contact lens was placed in a new UV cuvette with PBS, and the central area of the lens was illuminated by the light source. The incubation cycles continued until the contact lens sample reached a constant intensity. The emission spectrum color lines represented each incubation cycle. Emission spectrum and time-dependent intensities of F-Lys lens measured using a spectrofluorometer (**B**). The inserted FLIM images show the F-Lys on the Lot A lens at 0 min and 75 min.

We used silicone hydrogel (SiHG) lenses, which are easily available and are known for their higher oxygen permeability (DK). The increased DK value is due to the presence of silicone-rich areas, as silicone facilitates oxygen diffusion. Moreover, because of their higher silicone composition, SiHG lenses exhibit significant permeability to water and ions present in tears [63,64,65,66]. This permeability is due to the presence of a semi-interpenetrating polymer network (IPN) with continuous channels of essentially pure water or tear fluid from the front to back surfaces of the lens (Table S1 and Figure S3). The optical purity of the lenses is due to the size of the water channels being smaller than visible wavelengths, while the structure of the IPN indicates the presence of interface regions transitioning from non-polar to polar within the lens [63,64,65,66]. We have previously developed two methods to attach fluorophores to SiHG lenses, such as (i) the hydrophobic regions of SiHG lenses by covalently linking ion-sensitive fluorophores (ISFs) to long-chain hydrocarbons (i.e., C16 or C18), and (ii) conjugating ISFs to the highly charged polymer poly-L-lysine, which binds rapidly to SiHG lenses [63,64,65,66]. For both methods, the probes cannot be washed out of the lenses after weeks in buffer solution, and the binding seems to be irreversible in aqueous solution. In contrast to the SiHG lenses, the probes washed out quickly from HG lenses. SiHG lenses are made using polymers that contain free carboxyl groups, giving the lens a net negative charge [63,64,65,66]. CLs become coated with proteins after insertion into the eye and localization in the central aqueous layer of the tear film occurs when worn by patients. Human serum albumin (HSA) and Lys are the two proteins that are most often found in tears [74,75]. The binding of Lys molecules to the surface of the lenses was facilitated by the positive charge of Lys at a pH of 7. Notably, Lys is a protein with a size of ~14.3 kDa consisting of 129 residues [76,77]. A simple dip experiment was conducted to examine the binding rates of Lys molecules to the lenses at short time intervals (Figure 2A and Figure 3). Figure S4 shows the absorbance and emission spectra of Lys in PBS buffer and on CLs. Lys includes six tryptophan residues, which generally exhibited absorbance at 280 nm and fluorescence emission at approximately 350 nm [76]. The absorbance signal of Lys on the CLs was minimal, whereas the emission signal was clearly observed. This indicates that Lys could generate a thin multilayer on the surface of the CLs. These findings validate the existence of Lys on the CLs.

### 3.2. Tear Flow Across an Eye and Contact Lens

The use of contact lenses to detect biomarkers will require an understanding of tear flow across the eye. Tears originate in the lacrimal and Meibomian glands and flow across the eye to be collected in the lacrimal sac (Figure 1A). Basal-level tears are produced at a rate of 1 to 2 microliters (µL) per minute, and the total volume of tears in one eye is replaced in 7 to 9 min. The tear film is a complex structure that protects the cornea epithelium. The tear film is hydrophobic near the corneal surface and the outer surface is covered with a lipid layer to reduce the rate of water evaporation. Physical contact with the eye results in a rapid increase in reflex or emotional tears, which has a different composition than basal tears. The remarkable stability of tear films is demonstrated upon the insertion of a CL. The contact lens is quickly localized in the aqueous region of the tear film between the outer layer of lipid and water and the corneal epithelium. This results in two aqueous layers called the PLTF and PoLTF [66].

At this early stage of tear biomarker research, it is not practical to create a precise physical model for tear flow. The PLTF and PoLTF have been reported to be 2 to 8 µm thick. Reported microfluid devices have a minimum sample thickness of 25 µm for completely flat surfaces [78]. The tears can flow along different pathways, above and below the diameter of the lens, and above and below the lens in the PLTF and PoLTF. It would be difficult to create a physical model for tear flow. For these reasons, we decided to use a much simpler method to measure protein binding to contact lenses. We used a sample dip rinse and fluorescence measurement to detect the binding of each protein layer to the contact lenses (Figure 2A and Figure 3).

The protein binding to Lot A lenses can be effectively accomplished by this simple dipping technique. Lot A lenses were immersed in a phosphate-buffered saline (PBS) solution containing a concentration of 2.25 µM of fluorescein isothiocyanate (FITC)-labeled lysozyme (F-Lys) (NanoCS, Boston, MA, USA) for a period of 3 min (Figure 2A). Then, the lens was subjected to a 3 min rinsing process using PBS. The lens was then carefully inserted into a cuvette that was filled with PBS (Figure 3A). The contact lens was restrained diagonally in the cuvette, and we attempted to place the lens in the same position after each dip and rinse step. The procedure was replicated systematically at consistent intervals of 3 plus 3 min until the intensity of the emission spectrum and/or intensity reached a steady state (Figure 3B). The ISS laser scanning confocal microscope was employed to image the emission of the entire lens before and after the labeling steps. The Lot A lenses were non-fluorescent before labeling and uniformly fluorescent after the labeling steps (insert, Figure 3B). The lenses were labeled on both the inner and outer surfaces. Similar results were found for the Con A lenses.

**Figure 4:**
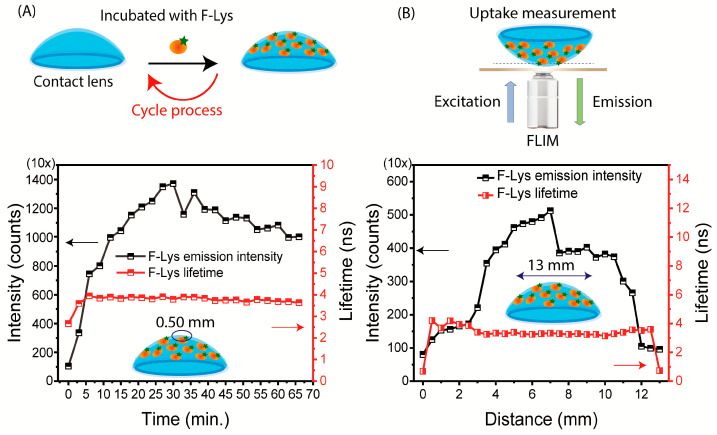
(**A**) Time-dependent intensities and lifetime for F-Lys binding to the contact lens. The diameter of the central area was near 0.50 mm. (**B**) The intensity and lifetime measurements were obtained using the F-Lys lens, moved at intervals of 0.50 mm across the contact lens. The objective focus was adjusted for the locations of the lens. The acquisition time was 20 µs for both intensity counts and decays.

For clarity, we note that the Lot A lens was dipped multiple times into the same 2 mL solution of F-Lys. There was some decrease in volume after each step. For this reason, the emission intensities in Figure 3 may be affected by the depletion of F-Lys in the labeling solution. As discussed previously, point-of-care testing requires fluorescence measurements that are independent of total intensity or intensity fluctuations [79,80]. The lifetime of F-Lys was measured in the central region during the binding process (Figure 4A). After complete binding, measurements were made at 0.5 mm increments along the x-axis across the contact lens (Figure 4B). In both cases the intensity varied by more than 10-fold, but the lifetime remained completely constant. The details of emission intensity counts and lifetime data are provided in Table S2 and Figure S5. This result indicates that the lifetime measurements will not require precise lens placement in an external reading device. If needed, a reference fluorophore could be added to the SiHG lens for wavelength–ratiometric measurements and/or for the determination of the amount of protein bound to the lens.

### 3.3. Binding of Antibodies to the Contact Lens

**Figure 5:**
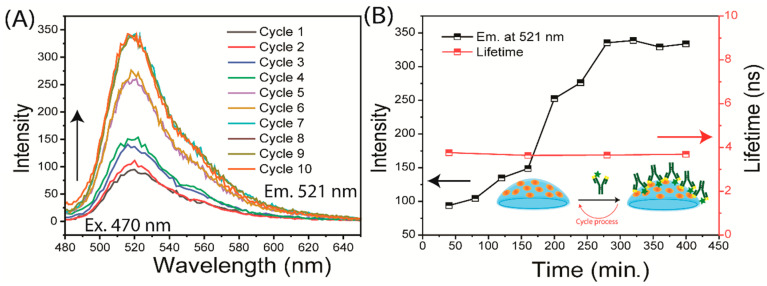
Time-dependent measurements of F-anti Lys binding to a Lys-coated lens. The measurements were stopped once a constant intensity was reached. (**A**) Emission spectra (the emission spectrum color lines represented each incubation cycle); (**B**) intensity and lifetime.

In this initial experiment, we considered lysozyme to be the capture protein. The next step was to determine whether a second protein could be captured on the CL. For this purpose, we used unlabeled lysozyme as the capture protein and fluorescence-labeled anti-lysozyme (F-anti-Lys) as the target protein or the biomarker protein. The Lys-coated lenses were first washed for four hours in PBS and then exposed to a 2 µg/mL solution of F-anti-Lys for 40 min (Figure 2B and Figure 5). After eight cycles of binding and rinsing, the emission intensity of the F-anti-Lys reached a limiting intensity. The lifetime of the F-anti-Lys remained constant throughout the binding process (Figure 5).

**Figure 6:**
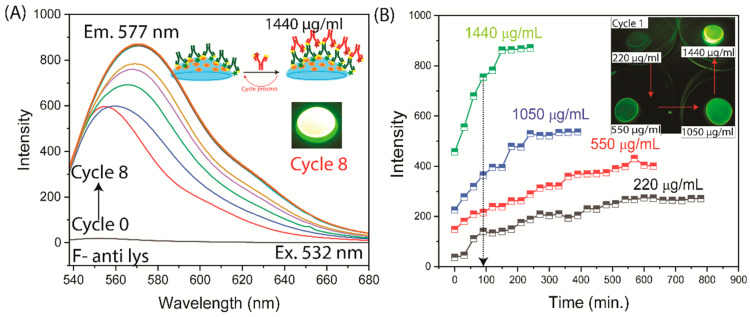
Time-dependent measurements of detection Ab (Rh-Anti IgG) binding to a F-Anti-Lys-coated contact lens. The measurements stopped once a constant emission intensity was reached. (**A**) Emission spectra (the emission spectrum color lines represented each incubation cycle); (**B**) intensity. The insert shows the emission image of the lens after cycle 1 for each Rh-Anti IgG concentration.

We used this same lens, coated with Lys and F-anti-Lys, to determine whether the Rh-anti-IgG detection Ab would bind to the lens with two protein layers (Figure 2C and Figure 6). In this experiment, the Rh-anti-IgG was considered the detection Ab to be used after the lens is removed from the patient. For this experiment, we used an excitation wavelength of 532 nm to avoid fluorescein excitation, but there was still a contribution from fluorescein, which has an extinction coefficient of about 2% of its maximal value of 1600 M^−1^ cm^−1^ compared to 90,000 m^−1^ cm^−1^ at its absorbance maximum. The amount of background from the F-anti-Lys varied in similar experiments, but the Rh-anti-Ig detection Ab could always be reliably measured. The F-anti-IgG background is the reason for the emission maxima shift shown in Figure 6 and Figure S6. The rate of Rh-anti-Lys binding was strongly dependent on its concentration (Figure 6B). As expected for a biomolecular reaction, the binding ratio (from the initial data points) was approximately 5.6-fold faster for a 6.3-fold increase in the Rh-anti-IgG concentration.

**Figure 7:**
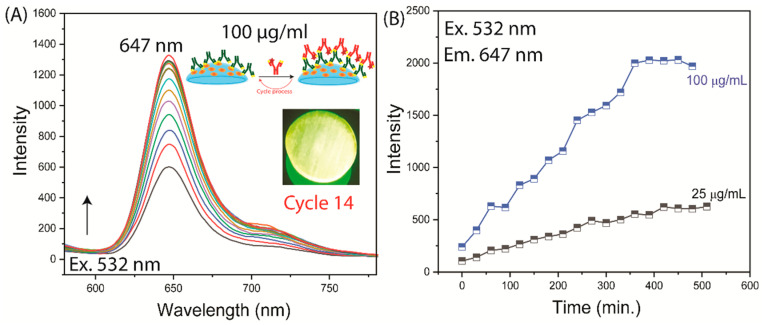
Time-dependent measurements of AF-Anti IgG binding to an Anti-Lys-coated contact lens. The measurements stopped once a constant emission intensity was reached. (**A**) Emission spectra (the emission spectrum color lines represented each incubation cycle); (**B**) intensities. The insert shows the emission image of the lens after incubation with AF-Anti IgG (Cycle 14). Time-dependent measurements were taken at 50 µg/mL but are not shown.

To avoid the fluorescence background from fluorescein, we made two changes to the experiment. The F-Lys-IgG was replaced with unlabeled Lys-IgG. Additionally, we used a detector Ab labeled with Alexa Fluor 647, which has longer absorption and emission maxima than rhodamine. Binding of the detection Ab could now be observed without any unwanted background fluorescence (Figure 7 and Figure S7). The inserts in Figure 7 and Figure S7 show that the lenses were labeled with the detection Ab. The emission intensity and lifetimes of AF-anti-IgG across the contact lens were examined; the intensity changed in different regions of the lens, while its lifetime remained stable (Figure S8). The primary and secondary antibody conjugates demonstrated comparable lifetime values, confirming their stability and resistance to interference. However, emission intensity provides a straightforward and measurable way to evaluate protein levels and detection limits, while lifetime analysis serves as a dependable additional method to confirm the presence of the specific biomolecule of interest.

The concentration of protein in the eye and its absorption by CLs significantly influence detection sensitivity. In the present study, we used Lys concentrations of 1 mg/mL and 0.1 mg/mL on the surface of the CLs. When the lenses were incubated in 0.1 mg/mL Lys for durations of 30 to 120 min, a strong Lys signal was consistently detected from the initial cycle (Figure S9), despite variations in antibody concentration. Furthermore, the present method of Lys detection is lower than the physiological Lys concentration in tears. Long-term lens wear can enhance protein accumulation, but it has no effect on the final measurement in our method.

## 4. Discussion

The results described above strongly suggest that capture immunoassay can be performed using SiHG contact lenses. As presently designed, the biomarker contact lens (BM-CL) will be placed on the patient’s eye for a given period of time, removed, and then the emission measured with a nearby fluorescence spectrofluorometer. Many such devices have been reported using cell phones or other array detectors [81,82,83], and point-of-care measurements are readily available. The excitation light will most likely come from an external source such as electroluminescent (EL) panels, which are available with single-color or multi-color output such as those used in computer servers. Such panels are available at a low cost, typically less than USD 10. Cell phone detectors will require careful setting of the lens and detector to override the automatic features using the cell phone controls or by using dedicated apps [84,85]. Because the BM-CL will be removed from the patient prior to measurement, other types of probes can be used for detection. The so-called time-resolved immunoassay can be performed using long-lifetime lanthanides bound to the detection antibody. In this case, the incident light is turned off prior to integrating the long live lanthanide emission [86,87]. Enzyme-amplified ELISAs could also be used [88]. Recently, an all-organic nanoparticle has been described with decay times of 200 to 300 ms [89]. The authors designed a pocket size reader, without optical filters, costing less than USD 20.

Tear fluid contains disease-specific biomarkers with diagnostic capabilities. For instance, some significant biomarkers include (i) Tau, amyloid-β-42, and lysozyme-C associated with Alzheimer’s disease; (ii) peroxiredoxin-6 and α-synuclein linked to Parkinson’s disease; (iii) lactotransferrin and lipophilin-A relevant to diabetic retinopathy; (iv) zinc-alpha-2 glycoprotein-1, prolactin, and calcium-binding protein-A4 pertaining to Graves’ orbitopathy; and (v) interleukin-6, interleukin-1β, TNF-α, and MMP-9 involved in dry eye disease [40,44,90,91,92]. These biomarkers demonstrate the diagnostic importance of tear analysis in systemic and ocular conditions.

**Figure 8:**
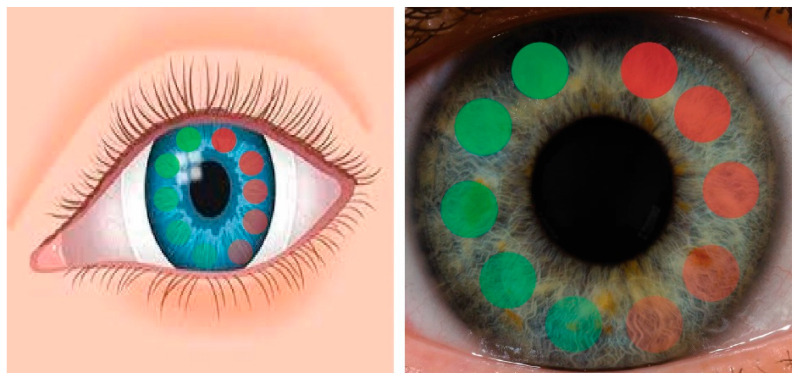
Contact lens used to measure MMP-9 over an expected concentration range. Green, contact lens with variable surface concentration of anti-MMP9. Red, control spots with ranges of intensity.

Our approach to basal tear biomarker assays has many potential advantages. While biomarkers can result from disease in any part of the body [93,94,95,96,97], the initial use of this BM-CL will be for the detection and diagnosis of ocular surface disease (OSD) (Figure 8). Many OSDs are known, including dry eye disease (DED), inflammation of the eyelid (blepharitis), inflammation due to allergies (conjunctivitis), membrane gland dysfunction (MGD), glioma, or injury to the delicate corneal surface.

The usefulness of our BM-CL can be seen by tests currently conducted in an ophthalmology office. The OSD mentioned above resulted in elevated levels of matrix metallprotease-9 in tears (MMP-9), which is one of the many MMP9s responsible for tissue remodeling [93,94,95,96,97]. Any damage or inflammation of the cornea results in high levels of MMP-9, which digests the tight junctions and allows tears to mix with the stroma. At present, the MMP-9 in tears is measured in two ways. Both methods require a sample of basal-level tears, which are collected with a capillary tube. Because of the small volume, the samples are sent out for amplified assays such as ELISA or PCR. The results are not returned for several days or a week, and are therefore not useful in a typical ophthalmology office visit.

The use of the SCL for clinical testing will require a decision regarding how long the lens should be left on the eye. This will depend on the concentration of MMP-9 or other target analytes, the flow rate of the tears, and the efficiency of MMP-9 binding during passage over or under the lens. At this time, the fraction of tear fluid that passes across the lens (by the PLTF or PoLTF) or around the control lens is not known. Mixing occurs in the PLTF and PoLTF dye upon blinking [66,98]. Commercial contact lenses can have different surface treatments [99,100], which can affect binding efficiency. We expect the biomarker binding efficiency to be high for several reasons. In other tests with flow devices, the binding efficiency was found to increase in thinner or smaller spaces [101,102,103]. This effect is probably the result of transitional diffusion in the film, which results in repeated collision with the CL surface. Another potential advantage of the SCL is the highly sensitive detection of low-concentration biomarkers. For example, if the lens remains on the eye for 30 min, the tear volume will be exchanged about four times, and a low-concentration biomarker could be collected from a large volume of tear flow.

In summary, we have demonstrated that a three-layer protein capture assay could be accomplished with a commercial SiHG contact lens. At present, we imagine the SCL will be removed from the eye and then exposed to the labeled detection Ab. Since this concept has not been tested in vivo, there is much to learn about the performance of these lenses. Point-of-care measurements, whether in vivo or after lens removal, are most accurately performed by a wavelength–ratiometric or lifetime-based measurement. This could be accomplished by the placement of a reference fluorophore in the SiHG to serve as an intensity or lifetime standard. SiHG lenses are known to bind hydrophobic fluorophores or fluorophores with hydrophobic side chains. We are hopeful that these results contribute to the increasing use of tears for diagnostic testing.

## Data Availability

Data supporting the findings of this study are available from the corresponding author Joseph Lakowicz upon request.

## Supplementary Materials

The following supporting information can be downloaded at https://www.mdpi.com/article/10.3390/bios15050326/s1: Figure S1: Typical smart contact lenses. Figure S2: Biomarker contact lens. Table S1: Technical data for contact lenses. Figure S3: Schematic for two silicone hydrogels. Figure S4: Lysozyme absorbance and emission spectra. Table S2: Emission intensity and lifetime value. Figure S5: FLIM lifetime measurement. Figure S6: Time-dependent measurements of detection Ab (Rh-Anti IgG). Figure S7: Time-dependent measurements of AF-Anti IgG. Figure S8: Confocal microscopy and FILM. Figure S9: Time-dependent incubation of lysozyme.
