# Supplementary material for: On the Possibility of Fluorescent Capture Immunoassays on a Contact Lens
Source: Biosensors (Basel). 2025 May 20;15(5):326. doi: 10.3390/bios15050326 (PMC12110756; doi:10.3390/bios15050326)
Supplement: Supplementary file 1 [file biosensors-15-00326-s001.zip › biosensors-3606449-supplementary.pdf]

Supplementary Materials

On the Possibility of Fluorescent Capture Immunoassays on a Contact Lens

Kundan Sivashanmugan [1], E. Albert Reece [2] and Joseph R. Lakowicz [1,*]

[1] Center for Fluorescence Spectroscopy, Department of Biochemistry and Molecular Biology, University of Maryland School of Medicine, 721 West Lombard St., Baltimore, MD 21201, USA

[2] Department of Obstetrics, Gynecology and Reproductive Sciences, University of Maryland, School of Medicine, 655 W., Baltimore, MD 21201, USA

* Correspondence: jlakowicz@som.umaryland.edu

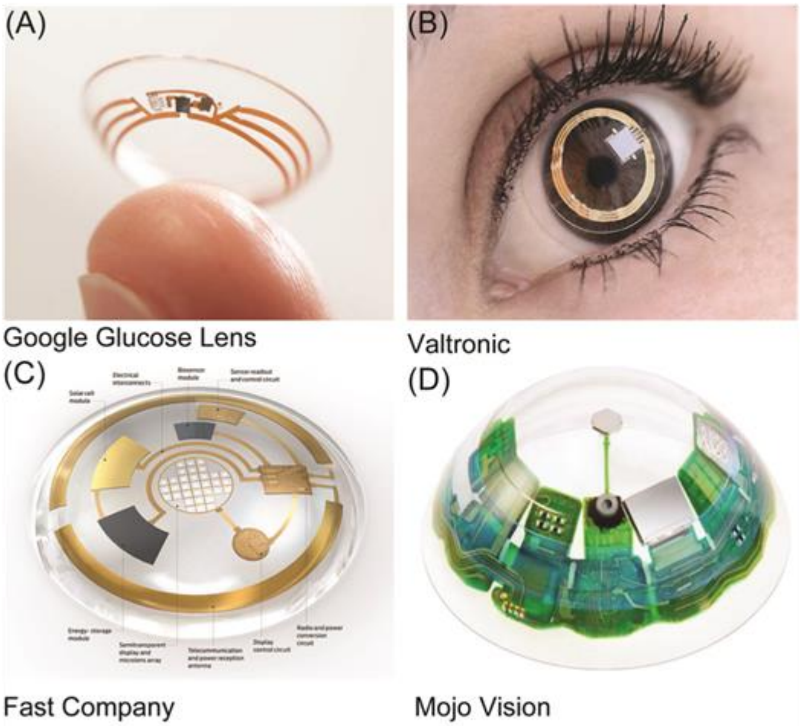

Figure S1. Typical smart contact lenses. (A) Google Glucose lens, (B) Valtronic lens to monitor for glaucoma, (C) Fast Company for image display and (D) Mojo Vision for augmented reality.

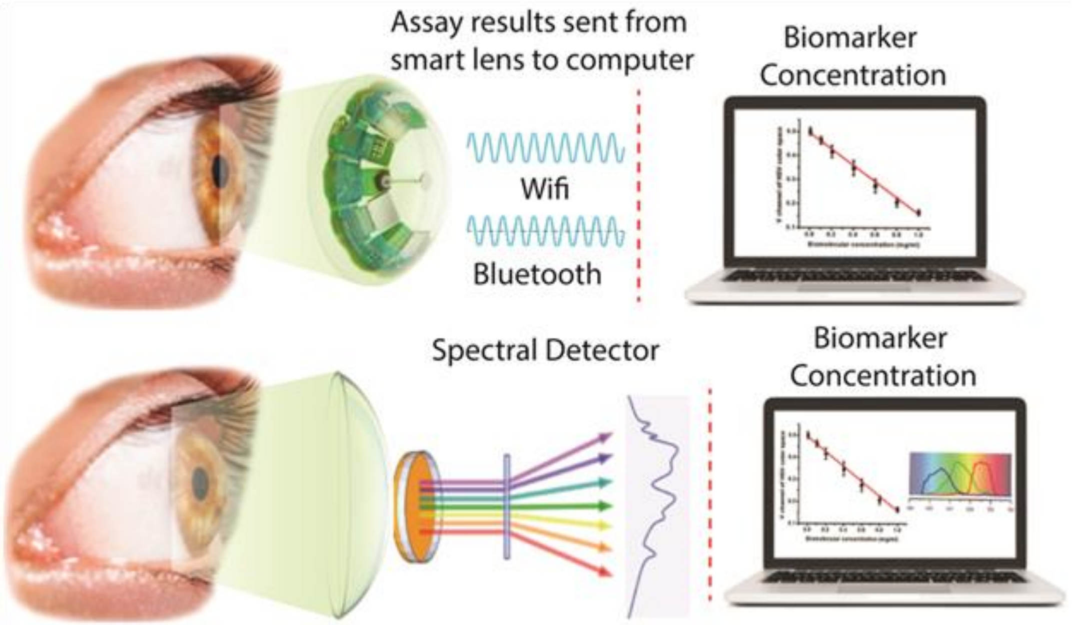

Figure S2. Top, biomarker concentration measured using a smart contact lens with embedded electronic components. Bottom, biomarker concentration measured using emission of BM-CL with electronics outside lens.

Table S1. Technical data for contact lenses.

Polymer	Trade Name	Manufacture	Wear Days	Water %	Dk (at -3.00D)
Comfilcon A (SiHG)	Biofinity	Cooper Vision	30	48	160
Lotrafilcon A (SiHG)	Air Optix	Alcon	30	24	175
Nelfilcon (HG)	Dailies	Ciba Vision	1	69	26

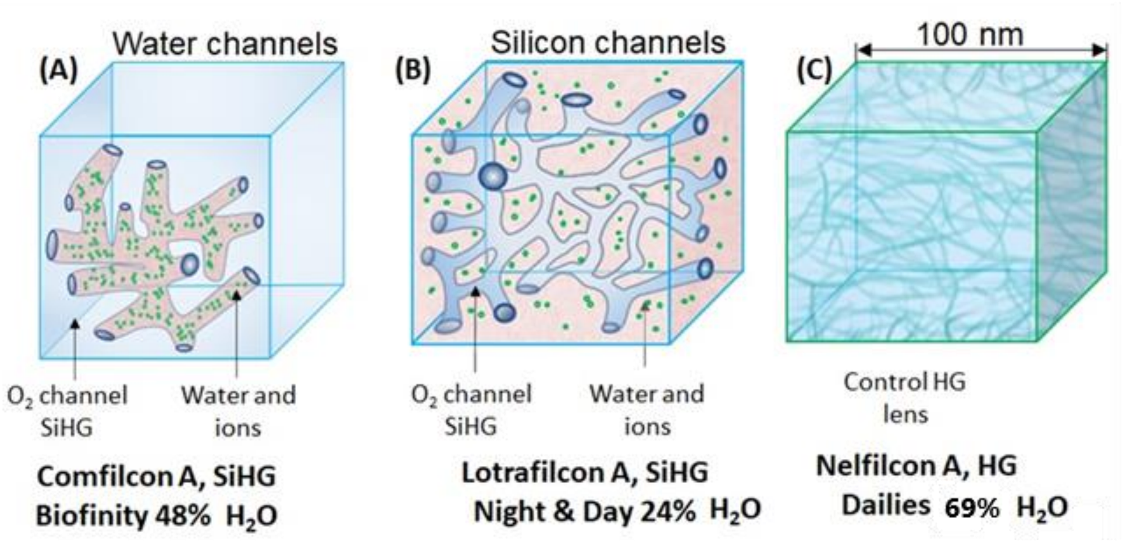

Figure S3. Schematic for two silicone hydrogel (SiHG) (A and B) and for standard HG contact lenses.

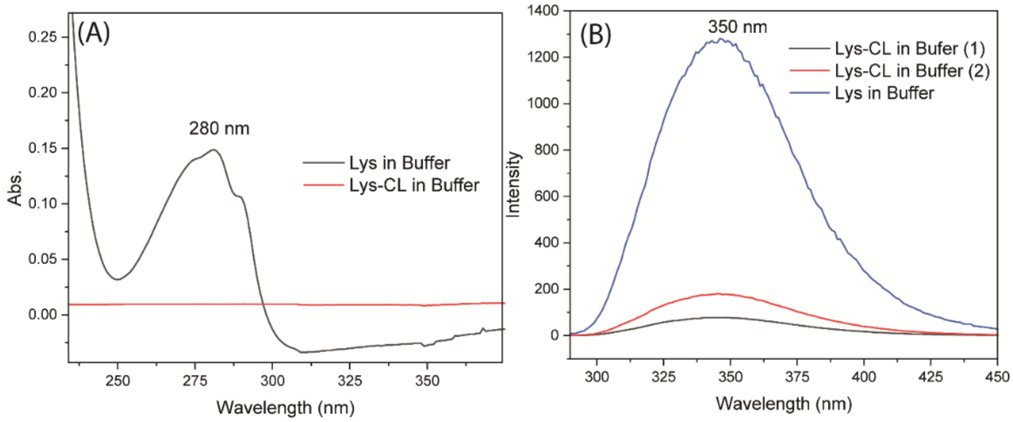

Figure S4. Absorbance (A) and (B) emission spectra of lysozyme in PBS buffer and on contact lenses. Note, Com A lens: Lys-CL in Buffer (1) and Lot A lens: Lys-CL in Buffer (2).

Table S2. Emission intensity and lifetime value for both CL center position and across CL positions (Figure 4).

CL center position (0.5mm)			Across CL positions		
Cycles (3 min.)	Emission intensity (counts)	Lifetime (ns)	Positions (0.5 mm)	Emission intensity (counts)	Lifetime (ns)
0	1043	2.665	0	805	0.688
3	3365	3.594	0.5	1250	4.199
6	7450	3.944	1	1535	3.704
9	8010	3.841	1.5	1560	4.184
12	9985	3.892	2	1655	3.944
15	10450	3.832	2.5	1720	3.851
18	11535	3.886	3	2210	3.4
21	12089	3.865	3.5	3545	3.253
24	12500	3.806	4	3950	3.339
27	13500	3.91	4.5	4120	3.296
30	13715	3.791	5	4620	3.393
33	11583	3.897	5.5	4735	3.268
36	13095	3.89	6	4800	3.249
39	11930	3.84	6.5	4920	3.302
42	11897	3.733	7	5125	3.333
45	11145	3.749	7.5	3855	3.301
48	11380	3.76	8	3910	3.243
51	11325	3.66	8.5	3900	3.299
54	10530	3.776	9	4030	3.235
57	10625	3.733	9.5	3725	3.245
60	10835	3.685	10	3825	3.139
63	9985	3.677	10.5	3745	3.296
66	10030	3.633	11	3010	3.393
			11.5	2655	3.568
			12	1050	3.512
			12.5	990	3.602
			13	960	0.733

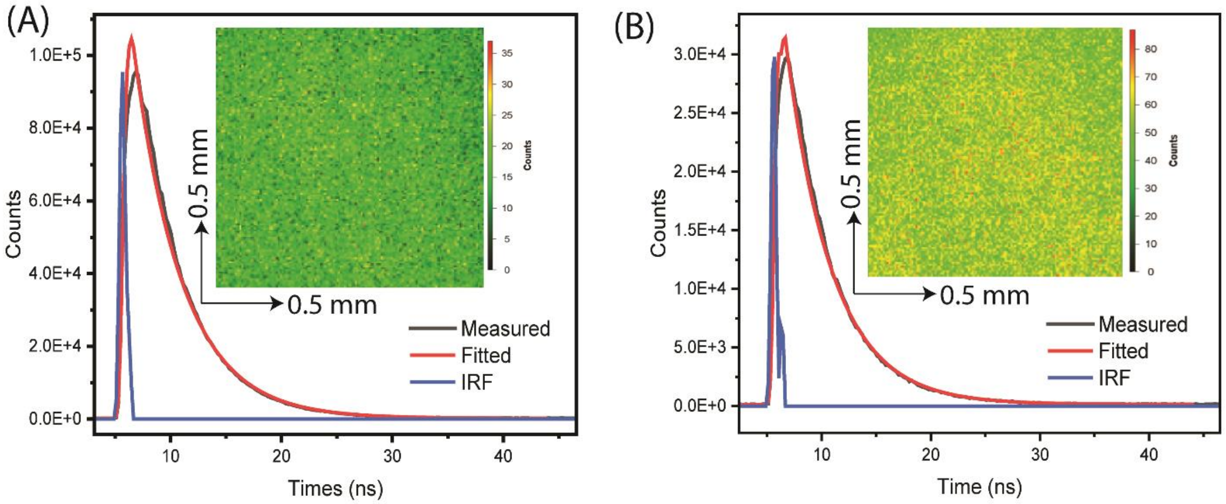

Figure S5. FILM was used to obtain measurements of lifetimes and inserted emission images in the center area of the CL and individual positions across the contact lens area (Figure 4A and 4B). The measured area was approximately 0.5 mm, and the 256 x 256 pixels were captured using a 20X objective lens. The lens sample was placed on a glass substrate and scanned along the X axis at intervals of 0.5 mm until 13 mm to gather the emission intensity and decay data. The focus of the objective lens was also adjusted based on the location of the contact lens.

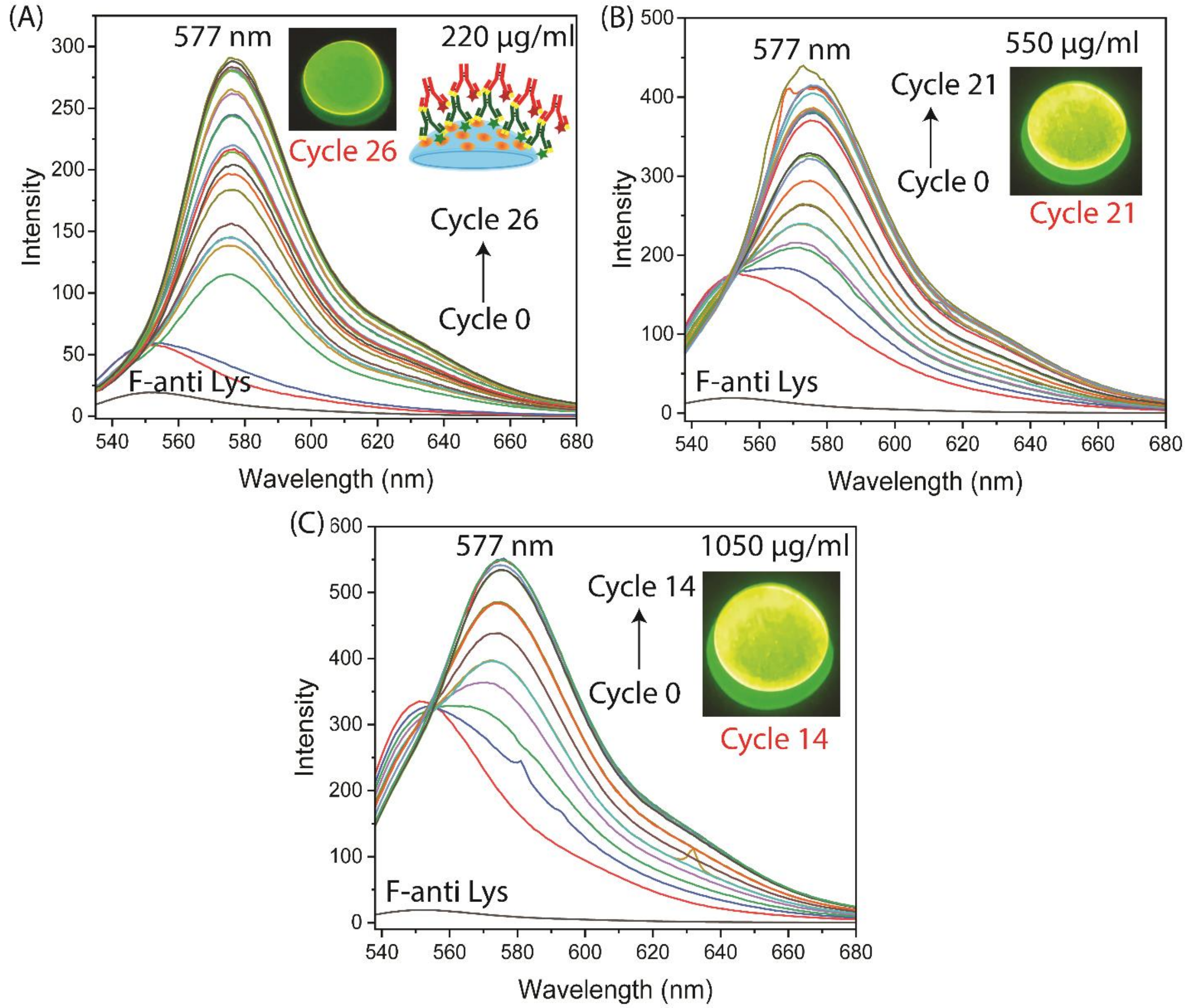

Figure S6. Time-dependent measurements of detection Ab (Rh-Anti IgG) binding to a F-Anti-Lys coated lens. The measurements stopped after reaching a constant of emission intensity, (A-C) emission spectra of the lens at various concentrations (220, 550, and 1050 µg/ml) of the detection Ab. The insert shows the emission images of the lenses after incubation with Rh-Anti IgG.

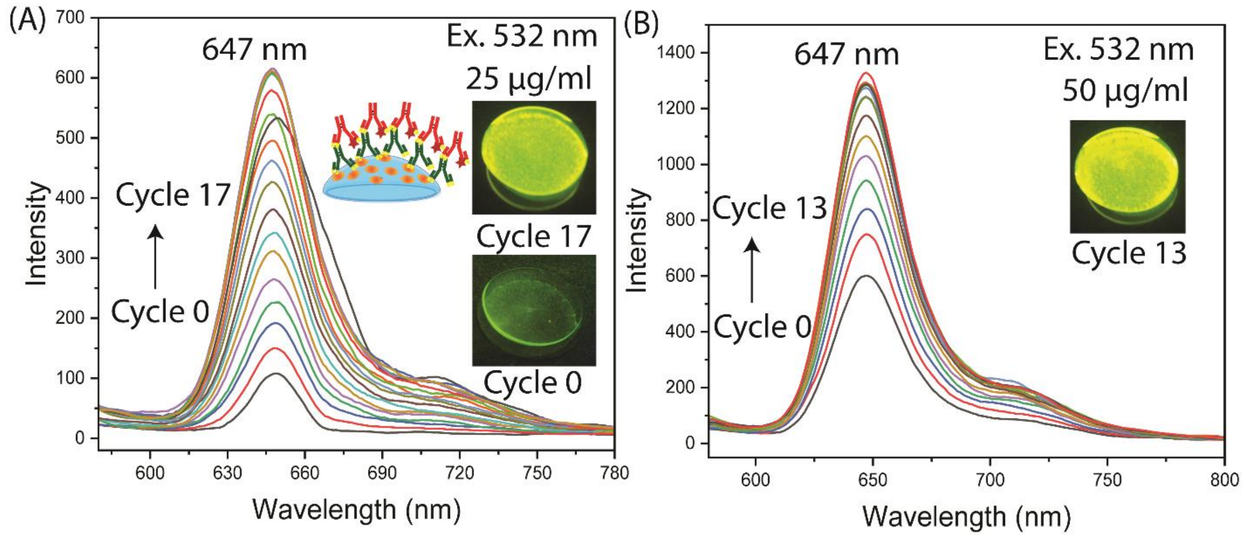

Figure S7. Time-dependent measurements of AF-Anti IgG binding with Anti-Lys on the contact lens. The measurements stopped at reaching constant of emission intensity (A-B). The image shows the AF-Anti IgG emission from the lens at the indicated cycle and AF-Anti IgG. The insert shows the emission image of the lens at after incubated cycle and AF-Anti IgG concentrations.

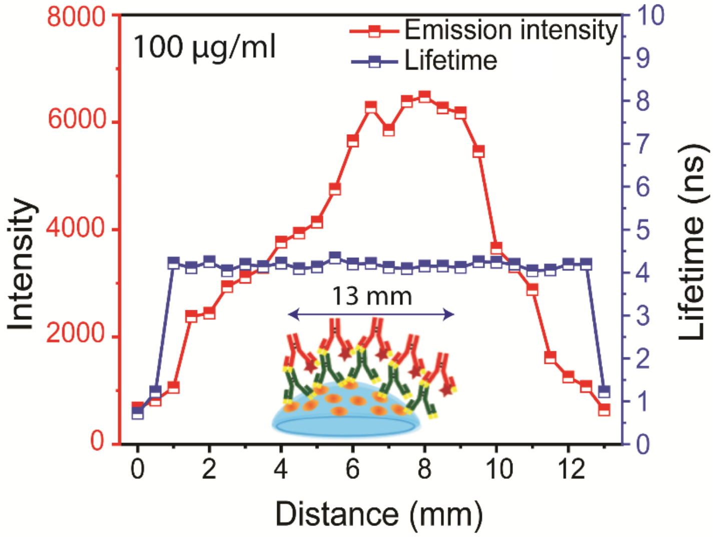

Figure S8. Confocal microscopy and FILM were used to measure of emission intensities and lifetimes for detecting AF-Anti IgG bound to Anti Lys coated on a lens (final incubated sample). The lens sample was placed to a glass substrate and scanned along the X axis at intervals of 0.5 mm until 13 mm to gather the emission intensity and lifetime. The focus of the objective lens was adjusted based on the location of contact lens.

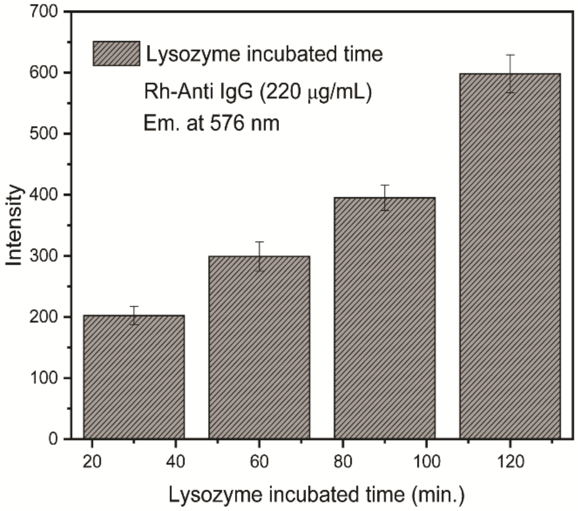

Figure S9. The time-dependent incubation of lysozyme (0.1 mg/mL and 30-120 min at 1st cycle) on contact lenses was detected using a Rh-Anti IgG (220 μg/mL).
